# The role of urodynamics in females with lower urinary tract symptoms

**DOI:** 10.1080/2090598X.2019.1589931

**Published:** 2019-04-04

**Authors:** Riyad T. Al Mousa, Nader Al Dossary, Hashim Hashim

**Affiliations:** a Department of Urology, King Fahad Specialist Hospital Dammam, Dammam, Saudi Arabia; b Bristol Urological Institute, Southmead Hospital, Bristol, UK

**Keywords:** Female urology, lower urinary tract symptoms, urodynamic, incontinence, overactive bladder, bladder outlet obstruction

## Abstract

**Objective**: To review the role of urodynamic studies (UDS) in females with lower urinary tract symptoms (LUTS), as LUT dysfunction is a common condition. The role of UDS was and continues to be vital in the assessment of such cases; however, utilisation is still debated amongst clinicians as to when and in which conditions it should be used.

**Materials and methods**: We conducted a literature review using the Medical Literature Analysis and Retrieval System Online (MEDLINE) search engine from year 1990 until August 2018, using the keywords: ‘female urology’, ‘lower urinary tract symptoms’, ‘urodynamic’, ‘incontinence’, ‘overactive bladder’, ‘bladder outlet obstruction’. We also reviewed the latest international guidelines related to the subject including: the International Consultation of Incontinence, American Urological Association, European Urology Association, and International Continence Society.

**Results**: Using >60 reference articles and international guidelines, our review showed that there is a trend of utilisation of UDS in females with LUTS.

**Conclusion**: UDS remains a valuable diagnostic test, which provides vital information to both the surgeon and patient prior to invasive treatment, with minimal morbidity.

**Abbreviations**: DO: detrusor overactivity; LUT(D): lower urinary tract (dysfunction); NLUTD: neurogenic LUTD; OAB: overactive bladder; P_det_Q_max_: detrusor pressure at maximum urinary flow; POP: pelvic organ prolapse; PVR: post-void residual urine volume; Q_max_: maximum urinary flow rate; UDS: urodynamic studies; (M)(S)(U)UI: (mixed) (stress) (urgency) urinary incontinence

## Introduction

The lower urinary tract (LUT) has two main functions: storage of urine at low pressure and emptying of urine at a convenient time and place. The organs of the LUT responsible for these functions are the bladder, urethra, and urethral sphincter. Any disturbance of the urinary storage or voiding phases, can lead to LUTS. LUTS is a term that covers symptoms that result from conditions and diseases affecting the bladder and the urethra. These are storage symptoms, which include the overactive bladder (OAB) syndrome (urgency, urgency urinary incontinence [UUI], frequency and nocturia), as well as pain and stress UI (SUI). Voiding symptoms, which include slow and/or interrupted stream, terminal, dribble hesitancy or straining. Suspicious symptoms, such as haematuria (blood in the urine) and dysuria (pain passing urine), may indicate a bladder tumour or UTI. Post-micturition symptoms include post-micturition dribble (or UI) and the sensation of incomplete emptying [1].

LUTS are very common and may affect individuals of all ages, in both genders. This term was introduced in 1994 to describe a patient’s symptoms without describing the cause of the symptoms [2]. LUTS have a negative impact on a patient’s quality of life from social, physical and psychological aspects, in addition to the high impact on health costs [3]. In women, the prevalence of LUTS and UI were reported to be up to 50% in some studies [4]. SUI represent ~50% of UI cases, UUI accounts for almost 10%, whilst mixed UI (MUI) represents almost 40% [4,5].

In a large European population-based survey of LUTS (EPIC study), 66% of women reported at least one LUTS, with nocturia being the most common at 54.5%, followed by UI (13.1%) and urgency (12.8%), with an overall prevalence of 11.8% having OAB syndrome [6].

The basic assessment of female patients with LUTS includes a focused history and physical examination including pelvic examination, completing a 3-day bladder diary and validated symptoms’ score and quality-of-life questionnaire, and urine analysis [7].

The international guidelines recommend that after a full basic assessment, conservative therapy including life-style modification, exercises, and the use of oral medications without the need to go for any invasive investigations, including urodynamic studies (UDS), should be initiated [8,9].

UDS is defined as a standardised functional assessment of the LUT. It is an objective measurement tool to measure LUT function/dysfunction. In good urodynamic practice, UDS allows the identification of the underlying cause of the LUT dysfunction (LUTD) and may lead to a better understanding of a related pathophysiology. Good UDS practice consists of appropriate patient selection, relevant test measurement, accurate interpretation, and critical analysis of results [10].

Despite its vital role and unique property as a functional test, the role of UDS in the assessment of female patients with LUTS continues to be a heavily debated subject. The question of the debate that always arises is when and in which cases should UDS be utilised.

To answer this, we reviewed published articles and recent international guidelines that discussed the role of UDS in female patients with LUTS. Uroflowmetry, as a simple non-invasive UDS test, is not included in our comparison, as it represents one of the basic evaluation tests and the focus is mainly on invasive filling cystometry and voiding pressure-flow studies.

**Table 1.** EUA 2018 guidelines on UI in adults (UDS recommendation).

Recommendations *(NB: Concerning only neurologically intact adults with urinary incontinence)*	Strength rating
Clinicians carrying out urodynamics in patients with urinary incontinence should: • ensure that the test replicates the patient’s symptoms; • interpret results in the context of the clinical problem; • check recordings for quality control; • remember there may be physiological variability within the same individual.	Strong
Do not use urethral pressure profilometry or leak-point pressure to grade severity of incontinence.	Strong
Do not routinely carry out urodynamics when offering treatment for uncomplicated SUI.	Strong
Perform urodynamics if the findings may change the choice of invasive treatment.	Weak
Urodynamics practitioners should adhere to Good Urodynamics Practice standards defined by the International Continence Society.	Strong

**Table 2.** EUA 2018 recommendation for UDS and uro-neurophysiology.

Recommendations	Strength rating
Perform a urodynamic investigation to detect and specify lower urinary tract dysfunction, use same session repeat measurement, as it is crucial in clinical decision-making.	Strong
Non-invasive testing is mandatory before invasive urodynamic is planned.	Strong
Use video-urodynamics for invasive urodynamics in neuro-urological patients. If this is not available, then perform a filling cytometry continuing into a pressure-flow study.	Strong
Use a physiological filling rate and body-warm saline.	Strong

**Table 3.** AUA/SUFU adult UDS guidelines.

Recommendations	Grade of recommendation
Clinicians may perform UDS in women when it is important to determine if obstruction is present	C
Clinicians may perform UDS when it is important to determine if DO or other abnormalities of bladder filling/urine storage are present in patients with LUTS, particularly when invasive, potentially morbid, or irreversible treatments are considered.	Expert opinion
Clinicians should perform pressure-flow analysis in patients with relevant neurologic disease with or without symptoms or in patients with other neurologic disease and elevated PVR or urinary symptoms.	C

**Figure 1.**
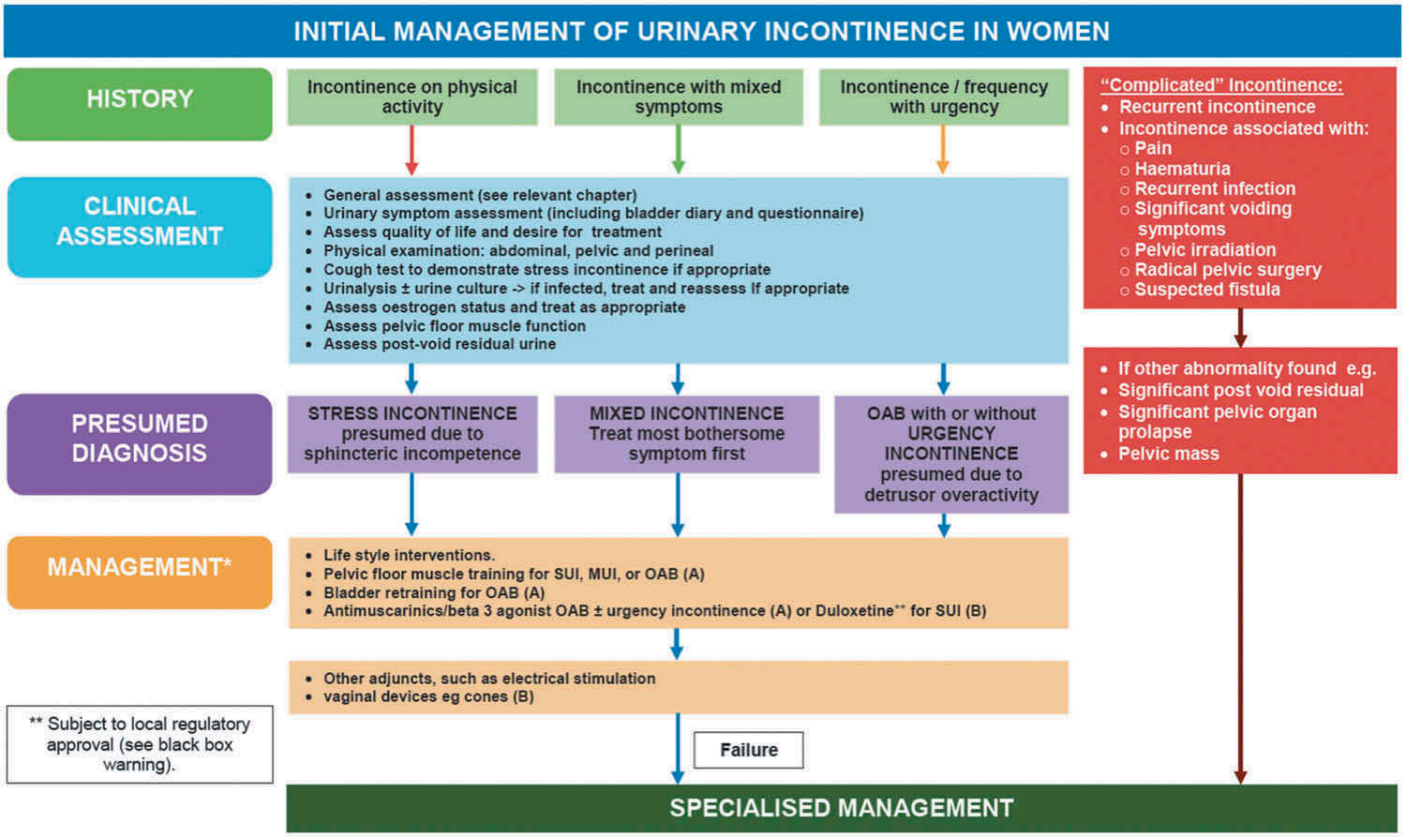
ICI 2016 initial management algorithm of female UI.

**Figure 2.**
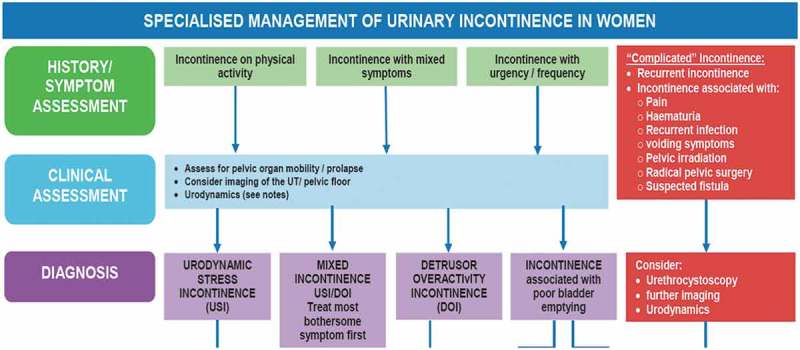
ICI 2016 specialized management algorithm of female UI.

**Figure 3.**
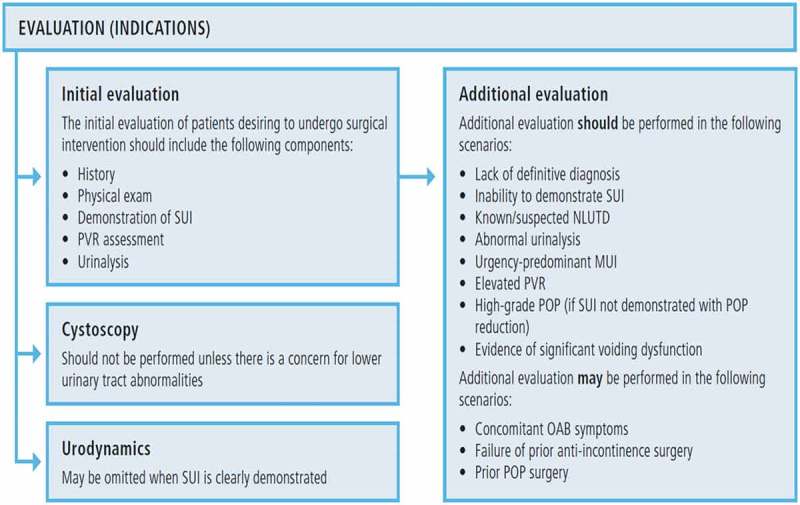
AUA/SUFU evaluation algorithm for female SUI.

## UDS: Basic and accepted general indications

According to the latest recommendations of the ICS and International Consultation for Incontinence (ICI) in 2016 [11], UDS have an overall accepted indication to assess LUTS function and LUTD, especially: when it may have a therapeutic consequence, and may change the therapeutic options or when it is performed as part of LUT surveillance or research. It is considered the ‘gold standard’ functional test to assess LUTS. Clinical applications include [11,12]:

- Better understanding of LUTS in terms of: available symptoms, other hidden symptoms, correlation between symptoms and prediction of impact on the upper urinary tract and other clinical consequences.
- Prediction of treatment outcome, side-effects of therapeutic options, or assessment of causes leading to failure of previous intervention.

During UDS, intra-abdominal and intravesical pressures are measured during filling and voiding through small catheters that are inserted in the rectum or vagina and bladder, and connected to the urodynamic machine through pressure transducers. By measuring intra-abdominal and intravesical pressures, the examiner can have a full picture of detrusor function and neural system integrity responsible for micturition [13].

## UDS use by LUTS type

### SUI

SUI is defined as the involuntary loss of urine during physical exertion, effort, coughing or sneezing in the absence of a detrusor contraction [14]. In some studies, SUI is reported as the commonest type of UI in women, with up to 86% reporting SUI. This can be either as pure SUI or in combination with other UI types (i.e., MUI) [15].

Conservative management is the first option of treatment before undergoing any surgical intervention. Due to the widespread use of mid-urethral slings over the last two decades, as a surgical treatment option for SUI [16], the number of SUI surgeries increased dramatically by 27% [17]. However, in recent years the overall number of procedures has declined due to regulatory warnings worldwide [18,19].

#### Complicated vs uncomplicated SUI

The use of UDS in patients with SUI can be categorised according to whether we are dealing with a complicated type of SUI or uncomplicated SUI. The ICS defines complicated SUI as: UI associated with pain, haematuria, neurological conditions, and recurrent UTIs, suspected voiding dysfunction, significant pelvic organ prolapse (POP), previous UI surgery, pelvic irradiation or surgery or suspected fistula; whilst pure SUI is referred to as uncomplicated SUI that is diagnosed mainly on basic clinical evaluation [20–22].

The role of UDS in complicated SUI prior to any surgical intervention is clearly defined in international guidelines [23–27] and all guidelines recommend the use of invasive UDS in these cases.

In pure SUI (uncomplicated SUI), we find a huge debate in the literature. Supporting the concept that ‘the bladder is an unreliable witness’ [28] many authors believe that UDS has an important role before any anti-incontinence surgery even for pure SUI. This belief is supported by the fact that symptoms do not always correlate with signs and hence UDS is important. In addition to that, pure SUI represents only a minority amongst female patients with SUI (5–10%) but still performing UDS in those patients would add value to the diagnosis, treatment modality, and surgeon confidence that ultimately might alter outcome, and most importantly give patients objective physiological evidence about their condition upon which they can make an informed choice about treatment.

The value of UDS was assessed in 3428 women with SUI. Only 8.9% patients were classified as having pure SUI and almost 20% of patients might not have needed surgery as the first line of treatment. Digesu et al. [29] in that study concluded that UDS provides useful information in the assessment of women with pure SUI.

In a large multicentre retrospective study from Italy, the prevalence of uncomplicated SUI cases that underwent UDS in six centres was investigated and showed that only 36% of those cases were classified as uncomplicated SUI, which again represent a minority in the study. The planned surgery was cancelled or modified in 19.2% of cases because of UDS findings. They concluded that UDS in uncomplicated SUI are useful and might alter management, whilst it is mandatory and unchallenged in complicated SUI cases [30].

Wang et al. [31] concluded in their study of 79 women with SUI that patients with preoperatively normal pressure-flow studies were more likely to have better quality of life and better pad test results after SUI surgery compared to those with abnormal pressure-flow studies (maximum urinary flow rate [Q_max_] <15 mL/s and detrusor pressure at maximum urinary flow [P_det_Q_max_] >20 cmH_2_O), which again supports the use of UDS in patients with pure SUI.

Alperin et al. [32] conducted a study that looked at the benefit of UDS prior to SUI surgery. Amongst 92 women who underwent sling surgery without evidence of *de novo* detrusor overactivity (DO), they found that 56% of women with a P_det_ >15 cmH_2_O during filling developed *de novo* UI compared to only 21% of women with a P_det_<15 cmH_2_O.

Documentation of associated asymptomatic DO in female patients with predominant SUI is important UDS information that adds value prior to SUI surgery. Other UDS findings that may govern future management plans include: detrusor underactivity, urethral function, and BOO [15].

On the other hand, those who do not support the use of UDS prior to SUI surgery base their opinions on systematic reviews, meta-analysis, and some multicentre studies that showed that UDS added unjustified costs, wasted surgeons’ and patients’ time, and delayed procedures without any clear reflection on final management outcome.

In the Value of Urodynamic Evaluation (ValUE) trial 630 women with predominant uncomplicated SUI were randomised to assess the value of UDS prior to surgery. Patients were randomised to office evaluation alone or office evaluation and UDS prior to surgery. The results showed that UDS increased the physician’s confidence in diagnosis but did not correlate with treatment success. They concluded that preoperative office evaluation alone was not inferior to evaluation with UDS for outcomes at 1 year (76.9% and 77.2%) [33].

In 30 Dutch hospitals, another multicentre cohort study, the Value of Urodynamics prior to Stress Incontinence Surgery (VUSIS), an embedded non-inferiority randomised controlled trial was conducted to assess the value of UDS prior to SUI surgery. They found that immediate sling surgery was not inferior to tailored treatment based on UDS in pure uncomplicated SUI [34].

Finally, in this regard, a meta-analysis and systematic review that was published in 2015 concluded that UDS does not add any value in women with pure SUI or MUI with predominant SUI, normal bladder capacity, and normal post-void residual urine volume (PVR) [35].

A recent expert consensus viewpoint on the value of UDS in female SUI was achieved in a review article that has been published recently. They concluded that extensive experience and observational studies have shown the danger of empiric management for SUI and strongly supported the value of UDS for the assessment of female SUI [36].

### OAB

OAB is defined as urinary urgency, usually accompanied by increased daytime micturition frequency and nocturia, with or without UUI, in the absence of UTIs, or other obvious pathology such as bladder tumour, stones or foreign body [35]. OAB is more prevalent in women, with overall prevalence increasing with age [6,35].

The consensus is that UDS is not indicated in patients with OAB prior to conservative or medical therapy. The main area of debate is that many clinicians believe that UDS is indicated in refractory OAB and only when initial therapy fails and it should be performed prior to any surgical intervention including minimally invasive procedures, such as sacral neuromodulation or onabotulinumtoxinA injection. They support this opinion with studies that showed that UDS is an invasive expensive tool, time consuming, and does not influence the initial management strategies [36,37].

Cho et al. [38] investigated the role of UDS in female patients with OAB. Clinical and urodynamic data of 163 women with OAB were analysed. They concluded that OAB symptoms were not useful for predicting presence of voiding dysfunction and for this UDS may be necessary for accurate diagnosis in women with OAB symptoms.

Many researchers believe that UDS is indicated only in patients with OAB symptoms after failure of primary therapy. They believe that UDS will not change the initial management strategies in such patients in addition to its cost and invasiveness [39].

Conversely, many others believe that UDS is still mandatory in female patients with OAB, as treatment based on symptoms alone may lead to under diagnosis of DO and storage symptoms that can be detected by UDS and that will ultimately alter the diagnosis and management plan [40,41].

One retrospective single-centre study confirmed that there is no association between subjective symptoms severity in patients with OAB, and bother and objective measures. This confirmed the role of UDS as an objective measure that is needed for better assessment [30].

Another multicentre study with good power confirmed that subjective symptoms assessment for women with LUTS was not sufficient to clarify the pathophysiology that caused the LUTS [42].

Although DO and OAB were considered as sharing the same characteristics, studies showed that they are not necessarily the same. Digesu et al. [39], in their study showed that only 27.5% of women with DO on UDS had OAB symptoms, whilst only 54% of women with OAB symptoms had DO on UDS, so they concluded that symptomatic diagnosis alone of OAB was not recommended for women with LUTS due to weak correlation between symptoms and UDS diagnosis. In another study, of the women with OAB symptoms, only 38.7% (63/163) had DO on UDS [38].

### BOO

In women, there is no clear urodynamic threshold values for the diagnosis of BOO compared to what is there in men. For this reason, the prevalence of BOO in women varies because of the lack of clear definitions or criteria for BOO. Generally, it has been found to range between 2.7% and 29% [43].

Blaivas et al. [44], introduced a nomogram for the diagnosis of BOO. A Q_max_ of <12 mL/s and a P_det_Q_max_ of >20 cmH_2_O were considered to represent an obstruction. Other researchers indicated that the presence of weak flow even in the absence of a high P_det_Q_max_ might represent a relative obstruction [45]. Chassagne et al. [42] used a threshold value of Q_max_ ≤15 mL/s and P_det_Q_max_ >20 cmH_2_O for the diagnosis of BOO. They reported 74.3% sensitivity and 91.1% specificity. With these thresholds values, they reported the prevalence of BOO to be 42.9% in women with OAB [42].

### Detrusor underactivity

Detrusor underactivity is a contraction of reduced strength and/or duration that results in failure to achieve complete bladder emptying or causes prolonged bladder emptying. It can be either idiopathic, which is most common, or due to neurogenic causes, previous pelvic surgeries, irradiation, or medications [46]. The prevalence varies between 3% in adult women [47] to 8.6% in women with OAB symptoms [1]. Again, those patients might have obstructive symptoms, high PVR, storage symptoms, post-micturition symptoms, or a combination of all. Because of this dilemma, without UDS, diagnosis cannot be reached in such cases [47].

Many indices have been used to assess patients with detrusor underactivity. The only definitive finding that is confirmed is the presence of an acontractile detrusor muscle, where there is no detrusor pressure recorded during voiding and the patient will void by straining only [1]. Other parameters that were discussed to assess such patients included: P_det_Q_max_ <30 cmH_2_O, Q_max_ <10 mL/s, and a bladder voiding efficiency (which is voided volume divided by voided volume plus PVR and <90% is suggestive of detrusor underactivity), reduced maximum cystometric capacity, and impaired compliance [47,48]. Therefore, UDS is important in such patients.

### Neurogenic LUTD (NLUTD)

In a systemic review of 49 studies, Musco et al. [49] clarified the mandatory role of UDS in assessing patients with NLUTD. They concluded that patients with spina bifida and spinal cord injuries have a higher risk of developing upper urinary tract dysfunction compared to those with multiple sclerosis. Major risk factors for upper urinary tract dysfunction were high detrusor leak-point pressure, reduced compliance, and lower functional bladder capacity. These risk factors clearly indicate the mandatory role of UDS in such patients.

### Post-menopausal women

In post-menopausal female patients, voiding dysfunction is a common problem and it is a major cause of impaired quality of life. Predisposing factors include ageing, oestrogen deficiency, increase in bladder fibrosis, and decrease in muscle fibres [50]. Choudhury et al. [51] evaluated the causes of LUTS in post-menopausal female patients and correlated symptoms with their UDS findings. In 100 patients, BOO, storage symptoms, and UI were the major LUTS findings. In all, 45% of patients were categorised as clinically obstructed and UDS showed that 62% of those had established BOO. They concluded that UDS was a necessary test along with physical examination in order to reach to a proper diagnosis and correct management plan.

## Non-conventional UDS

### Video UDS (VUDS)

The benefit of VUDS over conventional UDS has been questioned and highlighted in the literature and international guidelines. The aim of combined fluoroscopy with UDS is to combine the anatomical and functional information simultaneously. Information that can be utilised from VUDS includes: position of the bladder neck relative to symphysis pubis, bladder neck closure during rest and stress, detrusor–sphincter dyssynergia, VUR, presence of vesico-vaginal or urethro-vaginal fistulae, or presence of bladder or urethral diverticula. VUDS is recommended when conventional UDS fails to provide sufficient data to reach an appropriate diagnosis, especially in cases with neurological conditions and recurrent LUTS after failed surgery [52]. The ICS recommends VUDS as a reasonable option to assess complicated or recurrent female UI before any surgical intervention (Grade C recommendation) [53].

Similarly, the AUA in combination with Society for Urodynamic, Female Pelvic Medicine, and Urogenital Reconstruction (SUFU) considers VUDS as an option in patients with relevant neurological conditions, or in patients with LUTS (Grade C recommendation) [54].

### Ambulatory UDS

Ambulatory UDS is UDS that utilises natural filling of the bladder to reproduce a patient’s symptoms during regular daily activities. It is performed outside the urodynamic room and it allows patient to do their daily activities whilst performing the test, which helps to identify any activity that can provoke symptoms [55]. Ambulatory UDS may increase diagnostic accuracy [56]. However, a small number of studies have not shown enough evidence to support the use of ambulatory UDS over conventional UDS preoperatively [57]. Nonetheless, ambulatory UDS remains a useful second-line investigation in patients where appropriately conducted conventional UDS fail to reproduce the symptoms [58].

## International recommendations and algorithms on the use of UDS

All major international urological guidelines have fully explored and discussed the role of UDS in assessing patients with LUTS. To complete the picture of our discussion, we summarised related recommendations and algorithms as follows:

Table 1. European Association of Urology (EUA) 2018 guidelines on UI in adults (UDS recommendation).

Table 2. EUA 2018 recommendation for UDS and uro-neurophysiology.

Table 3. AUA/SUFU adult UDS guidelines.

Figure 1. ICI 2016 Initial management algorithm of female UI.

Figure 2. ICI 2016 Specialised management algorithm of female UI.

Figure 3. AUA/SUFU evaluation algorithm for female SUI.

## Conclusion

LUTS in female patients is a very common condition that may carry a serious negative impact on the social, financial, physical, and psychological well-being of patients and their families. The role of UDS continues to be a heavily debated subject for assessing female patients with LUTS. Nonetheless, UDS remains a valuable diagnostic test that provides vital information, to both the surgeon and the patient prior to invasive treatment, with minimal morbidity.
